# Worldwide human mitochondrial haplogroup distribution from urban sewage

**DOI:** 10.1038/s41598-019-48093-5

**Published:** 2019-08-12

**Authors:** Orsolya Anna Pipek, Anna Medgyes-Horváth, László Dobos, József Stéger, János Szalai-Gindl, Dávid Visontai, Rolf S. Kaas, Marion Koopmans, Rene S. Hendriksen, Frank M. Aarestrup, István Csabai

**Affiliations:** 1 Department of Physics of Complex Systems, ELTE Eötvös Loránd University, Pázmány P. s. 1A, Budapest, 1117 Hungary; 2 Department of Information Systems, ELTE Eötvös Loránd University, Pázmány P. s. 1C, Budapest, 1117 Hungary; 3 Department of Computational Sciences, Wigner Research Centre for Physics of the HAS, Konkoly-Thege Miklós út 29–33., Budapest, 1121 Hungary; 4 National Food Institute, Technical University of Denmark, Kgs., Lyngby, Denmark; 5 Viroscience department, Erasmus Medical Center, Rotterdam, The Netherlands

**Keywords:** Population screening, Genetic testing, Data acquisition, Haplotypes

## Abstract

Community level genetic information can be essential to direct health measures and study demographic tendencies but is subject to considerable ethical and legal challenges. These concerns become less pronounced when analyzing urban sewage samples, which are *ab ovo* anonymous by their pooled nature. We were able to detect traces of the human mitochondrial DNA (mtDNA) in urban sewage samples and to estimate the distribution of human mtDNA haplogroups. An expectation maximization approach was used to determine mtDNA haplogroup mixture proportions for samples collected at each different geographic location. Our results show reasonable agreement with both previous studies of ancient evolution or migration and current US census data; and are also readily reproducible and highly robust. Our approach presents a promising alternative for sample collection in studies focusing on the ethnic and genetic composition of populations or diseases associated with different mtDNA haplogroups and genotypes.

## Introduction

Due to the advances made in DNA sequencing in the last two decades, the general idea of obtaining the genetic code of every single person has become the hypothetical answer to many health-, demography-, forensics- and even history-related questions. The dubious legal, economical and ethical repercussions of this vision however render this approach presently unattainable. Before we reach individual level genome sequencing, an easier target may be community level pooled sequencing.

Health-related efforts would largely benefit from available genetic distribution data for local communities. Many breakthroughs have already been made in the discovery and study of diseases with the use of personalized sequencing in the hopes of enabling earlier and more accurate diagnosis, individualized intervention, guiding prevention strategies and monitoring the effects of treatments^1^. Effects of pandemics can also differ based on genetic background as certain gene variants may provide enhanced susceptibility or resistance to viral diseases^2–4^. These genetic determinants may be shared by larger phylogenetically related subpopulations and the advantages of population based screening of risk factors in symptomless individuals are immense and have been demonstrated by multiple studies^5,6^. Data collection however is problematic, as in order to realize prevention programs specifically tailored to smaller communities, the distribution of genetic variations in the local populations has to be first established. In principle, this would require the collection of genetic information from as many individuals as possible, which naturally raises many ethical and legal concerns, as well as the practical challenges of sample collection and analysis. With the increasing amount of health related information available, it is getting progressively more difficult to ensure confidentiality, especially because in many cases third-party access to the data is insufficiently controlled^7^. This matter is made worse by the fact, that personal genomic data are highly sensitive, as they contain information not only about the person taking the genetic test, but also about a broader group of people who are genetically related to the individual^8^. People can be discouraged from getting tested for certain diseases by fear of possible genetic discrimination by employers or insurance agencies based on the results^9^.

A closely related subject, the collection of data on ethnicity can be essential to study demographic tendencies, employment practices and opportunities, income distributions, educational levels, migration patterns and trends, family composition and structure, social support networks, health conditions of a population and optimal treatment and preventive measures^10–12^. Data on ethnicity are collected using a wide range of different methodologies and often rely on self-reporting, which makes standardization difficult^10,13^. The collection of data on ethnicity is also sensitive and the decision to collect and disseminate information on ethnic or national groups of a population has to be based on a number of considerations and national circumstances^10,13^.

Additionally, from a forensics viewpoint, the availability of a comprehensive database of the genetic distribution of populations worldwide would be highly beneficial. It has been shown^14^ that individual contributors can be detected in highly complex mixtures of human DNA collected from common surfaces even when only an extremely small portion of the mixture belongs to the person of interest. The statistical method used for such analysis is highly dependent on the availability of the allele frequencies of an appropriate reference population with similar ancestral components to the investigated mixture. Furthermore, whenever genetic identification is limited to a restricted part of the human genome due to DNA degradation in the accessible evidence, the local genetic distribution of the population could be used as an informative prior to fine-tune probabilistic models to determine the probability of a DNA match.

As an alternative to obtaining informed consent from many individuals, performing genome sequencing one-by-one and then pooling the data, samples collected from wastewater plants can be used as a pooled sample that may contain the same relevant information. From a surveillance point of view, urban sewage is attractive because it combines material from a large and mostly healthy population, which would otherwise not be feasible to monitor. In addition, analysis of *ab ovo* pooled samples does not require informed consent, thus limiting ethical concerns^15^, including those related to studying human DNA-sequences^16^. The nature of sample collection itself eliminates the need for further anonymization of the data, as it provides an inextricably anonymized mixture of genomic information about many individuals simultaneously.

In the COMPARE Global Sewage Surveillance Project, we initiated a global collection of urban sewage in 2016 with the purpose of determining the occurrence of antimicrobial resistance genes and infectious disease agents among the healthy human population using metagenomic sequencing^17^. Metagenomic sequencing of urban sewage allows not only the identification of disease causing agents, like bacteria and viruses, but also a lot of additional information present in the samples, which were not part of the original scope of the study. In the initial analyses, we observed that on average 0.2% of all reads could be assigned to humans^17^. This relatively small amount of human DNA is insufficient for the detailed profiling of genotype distributions across the populations but limiting the investigations to the mitochondrion can lead to meaningful results. It has been previously shown, that mitochondrial DNA is suitable to distinguish between fecal contamination of human, bovine, porcine and ovine in contaminated surface water samples^18^. Human mitochondrial DNA (mtDNA) is a short (16,569 base pairs (bp)) circular DNA present in multiple copies in a single human cell, which makes it easier to detect even in samples with low concentration human DNA content. Human mitochondrion is inherited only from the mother (though recent results indicate that this is not always the case^19^), and it has also been demonstrated that the inherence is clonal, thus mtDNA is transmitted from mother to offspring without germline recombination^20^. These make variants of the mtDNA eligible to track evolutionary patterns. The leaves of the human mitochondrial phylogenetic tree are the mitochondrial haplotypes, which can be assigned to mitochondrial haplogroups (the major branching points of the tree) based on their similarities.

Human mitochondrial DNA haplogroups and their distributions have been extensively investigated across different nations and geographical regions^21–29^, predominantly to uncover population origins and genetic structure. The accumulated information about the mtDNA haplogroup composition of ancient and current communities transformed genetic ancestry testing from an abstruse academic quest to a popular and common practice among the public. However, companies providing direct-to-consumer (DTC) ancestry tests have been criticised^30^ for supplying misleading information to their customers that can deeply affect their personal identities. One of the disadvantages cited is the limited amount of reference samples available in databases upon which inference of geographic ancestry is based. This aspect could be greatly improved by the global and up-to-date monitoring of the mitochondrial DNA composition of populations at different geographic locations.

In this pilot study, we aimed to identify traces of human mitochondrial DNA in the global sewage dataset and determine the local mtDNA haplogroup composition of the sewage catchment area.

## Results

### Coverage of the human mtDNA in the samples

Urban sewage was collected globally from 79 sample locations and sequenced using Illumina HiSeq obtaining an average of 120 million reads per sample (range: 8 to 398 million). For details see Hendriksen *et al*.^17^. The average coverage of the human mtDNA varied greatly among samples (Supplementary Figs 1 and 2b), and altogether 44 samples reached the limit of having a mean coverage of 10 or higher. These samples were further analysed.

**Figure 1:**
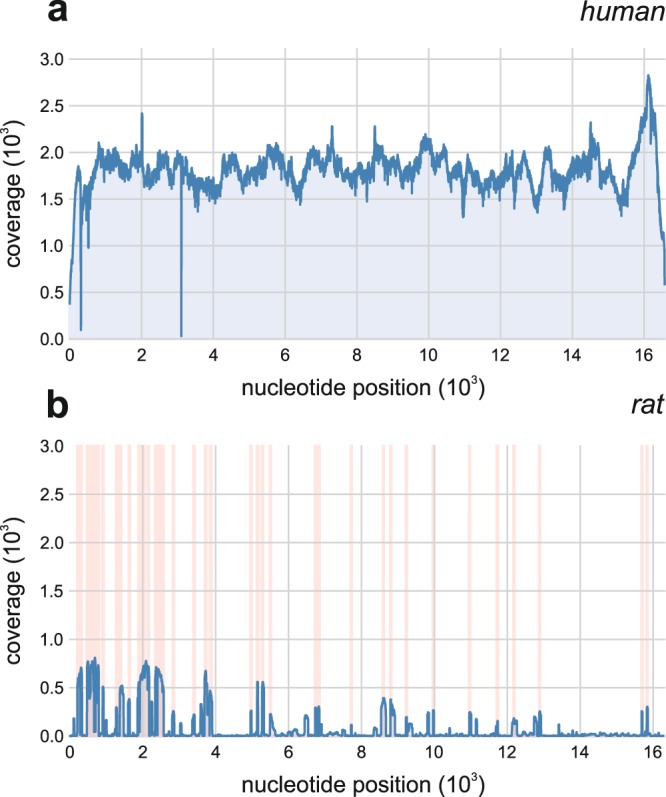
Coverage of the human and rat mtDNA in investigated samples. (**a**) Pooled coverage along the length of the human mtDNA for samples with an average coverage of 10 or higher. The approximately uniform distribution indicates that the chances of misaligned non-mtDNA reads are minimal. (**b**) Pooled coverage for the rat mitochondrion for the 44 analyzed samples (blue line). Light red vertical lines indicate genomic regions that are homologous to the human reference mtDNA. The measure of this quantity was defined on a binary scale for each genomic position as follows: if the given genomic position could be included in a section of the rat mtDNA with a sliding window method which section could also be (exactly) found in the human reference mtDNA, the position was deemed to be a homologous one. (The windowsize was chosen to be 19, as this is the default value used by the alignment algorithm as the minimal seed length.).

To ensure that the satisfactory average coverage along the length of the human mtDNA does not arise from false alignments of homologous non-human DNA segments to short regions resulting in on average high, but very uneven coverage, the pooled coverage was plotted for all the 44 analysed samples on Fig. 1a. The coverage of the mtDNA is fairly even in the investigated samples, the fluctuations do not exceed the known variation of next generation sequencing data^31^. This is also true when exploring coverage variations in single samples only, albeit with a much lower mean value (Supplementary Fig. 1.). This is however, not the case for other organisms, where besides a few local peaks in coverage, the rest of the mtDNA remains uncovered. This is demonstrated with an example of the rat mtDNA on Fig. 1b, where the peaks in coverage appear almost solely in those regions that are homologous to the human mtDNA. We have also plotted the number of reads aligned uniquely to the human and the rat mtDNA and the number of reads aligned to both on Supplementary Fig. 2a for each of the 44 samples. The exclusively human reads outnumber the reads unique to the rat mtDNA by a factor of 40 on average in samples with a mean coverage of 10 or more. Thus, we can conclude that the identified reads are indeed human and not the results of misalignment. No reads could be aligned to the human mtDNA in our three negative control samples (extraction kit controls, data not shown), thus human DNA contamination during the analysis process can be excluded.

### Unsupervised clustering with principal component analysis and t-distributed stochastic neighbor embedding

To confirm that the amount of human mtDNA found in our samples is sufficient for meaningful scientific conclusions to be drawn, we first tested whether the samples could be separated by unsupervised clustering algorithms according to their origin. Previous efforts^32^ have shown that principal component analysis (PCA) on the human mitochondrial genome can efficiently distinguish individuals based on their mitochondrial haplogroups.

**Figure 2:**
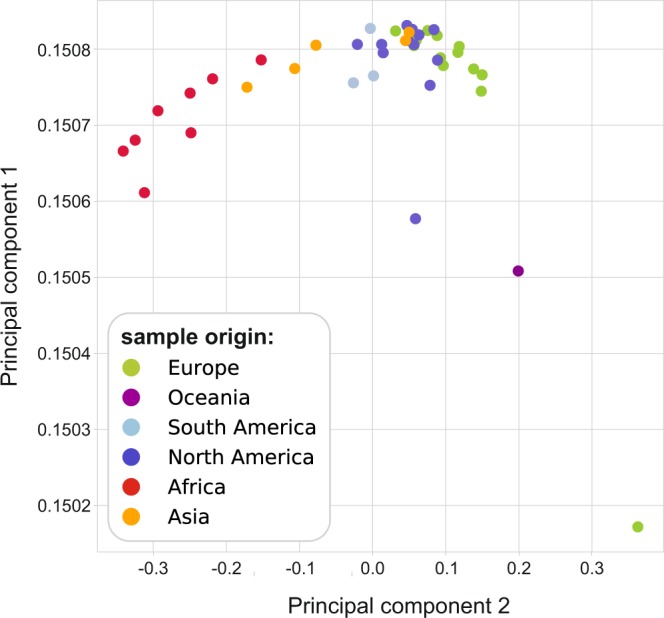
Principal component analysis of the 44 samples with average coverage higher than 10. Samples originating from different continents are marked with dots of different colors. PCA was carried out on the whole mitochondrial genome, using the most dominant base in each genomic position with one hot encoding. (The analyzed matrix for the 44 samples had a shape of (44, 4·16,569).).

We performed PCA on the 44 samples with an average coverage higher than 10 as a general exploration of sample features (see Methods for details). As human mitochondrial DNA haplogroups are distributed across geographical areas non-uniformly^25^, it is reasonable to expect that traces of human mitochondrion found in sewage samples would also differ between samples collected from various continents. To test this theory, we projected data from each sample to the subspace spanned by the first two PCA directions (percentage of variance explained 99.87% and 0.03% respectively) (Fig. 2). Samples originating from different continents were distinguished by different colors. As apparent on the figure, sewage samples from both Africa and Asia are remarkably well-separated from the rest of the samples. Samples from Europe and America tend to somewhat mix together, in line with our intuition and previous literary evidence^32,33^ of these continents having highly diverse populations due to migration. Outliers can however, also be observed on the figure for both Europe and North America. This is most likely due to the fact, that PCA uses a single consensus sequence to characterize each sample even though sewage samples contain mtDNA sequences from the mixture of a large population. Using only the most frequent base in each genomic position (a common practice for consensus sequence generation) artificially creates mixed mtDNA sequences fused together from different mtDNA haplotypes. An additional difference from the method described by Biffi *et al*.^32^ is that while they only used 64 tagging SNPs, in our analysis, the whole mitochondrial genome was incorporated to PCA.

As a basic confirmation of the validity of these results, we also performed t-distributed Stochastic Neighbor Embedding (t-SNE)^34^ on the samples, which technique uses a different algorithmic concept for dimensionality reduction. We were able to reproduce the above described clusters with this method as well (results not shown).

### Phylogenetic analysis

A slightly different approach that is fairly commonly used in human mtDNA analysis pipelines^35,36^ is the construction of phylogenetic trees, which aims at uncovering evolutionary relationships among samples. Given that the human mitochondrial phylogenetic tree has been extensively studied^29,35,36^, and human mtDNA haplogroups are defined as its major branch points, the inhomogeneous distribution of haplogroups among different geographical regions suggests that samples collected from the same areas should form clades on their own phylogenetic tree.

To explore the evolutionary relationships between samples, we constructed a phylogenetic tree of the 44 samples with an average coverage of 10 or higher based on the consensus sequences of these samples (Supplementary Fig. 3.). This method suffers from the same limitation as PCA due to using a single consensus sequence to represent a sample, rendering the results somewhat unreliable. Different tree constructing methods resulted in slightly different trees; nevertheless, the main conclusions remained the same. Samples originating from Africa formed a fairly distinct clade, while samples from Europe and America were slightly blended together but the robustness of the trees (see Methods for details) was very low, as expected from the nature of consensus sequence generation.

### Human mtDNA haplogroup composition of samples

**Figure 3:**
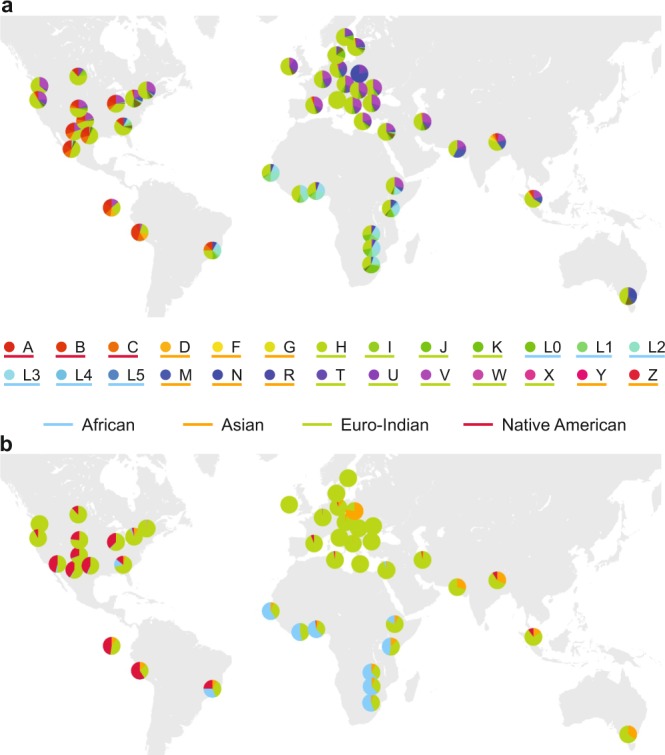
Mitochondrial DNA haplogroup composition of the 44 sewage samples with an average coverage of 10 or higher. (**a**) Mitochondrial DNA haplogroup composition of samples plotted at the site of the wastewater collection. Circle colors and colors of the pie charts correspond to specific haplogroups, while colors of the underscores indicate the four broad biogeographic ancestry categories. (**b**) Mitochondrial DNA haplogroup composition of samples using only the four broad biogeographic ancestry categories.

The above analyses proved that the human mtDNA content in our samples was sufficient to recover the expected tendencies, but the results were largely biased by the pooled nature of sample collection. To overcome the problem of using a single consensus sequence to represent a sample, we analyzed the samples separately and decomposed the aligned reads to differently weighted contributions of different mtDNA haplotypes with an expectation maximization approach^37^ (see Methods for details). The results of the analysis were plotted on a map as pie charts (Fig. 3). Color codes were selected to match those in Fig. 2 of Rishishwar *et al*.^25^ to allow an easy visual comparison. Our results show great agreement with the published results of Rishishwar *et al*.^25^, indicating that the mtDNA haplogroup composition of a given area can be accurately determined from trace human mtDNA detected in sewage samples.

### Comparison with available data

For a more direct comparison with published data of human mtDNA haplogroup compositions of different cities, we plotted mtDNA haplogroup pie charts of sewage samples along with available results of various studies on Supplementary Figs 4–7. (The full list of data sources used can be found at the end of Supplementary Information.) In Supplementary Fig. 7, the mtDNA haplogroup distributions of cities from the United States of America (US) were limited to ratios of mtDNA haplogroups belonging to the four broad biogeographic ancestry categories indicated by the underscores on Fig. 3. Results acquired using US census data can be directly compared to the inner pie charts of sewage samples.

In general, the mtDNA haplogroup composition of the urban sewage samples shows surprisingly great agreement with results previously obtained by careful sampling of specific populations in other studies. As anticipated, some differences do occur, given the extremely dissimilar natures of sample collection. Studies focusing on the evolutionary history of a particular population tend to single out very specific groups of individuals, while collecting wastewater in a given location results in a mixed sampling of all kinds of human mitochondrial DNA. As with any statistical result, the number of samples used by other studies and the relatively low coverage of the human mtDNA of the sewage samples can also contribute to the observed differences. Another factor of uncertainty is the decreasing precision of the software^37^ used for mtDNA haplogroup decomposition with the number of mitochondrial haplotypes to be recovered in the mixture. For three haplotypes, even the trace contributor (present in 5%) is correctly detected in most cases and only sometimes is it mistakenly identified as a closely related haplotype. Given that our analysis focused on mitochondrial haplogroups instead of specific haplotypes, the errors in the expectation maximization process are somewhat compensated. However, the possibility of increased ambiguity in complex mixtures should not be overlooked. It should also be noted that using only the broad continental ancestry groups for US cities is admittedly a compromise necessitated by the lack of mtDNA haplogroup distribution data specific to these cities. Although the classification is widely used in both scientific literature^38^ and commercial ancestry testing^39^, many studies have shown in recent years^40–44^ that continental-ancestry proportions often vary greatly among individuals sharing the same mtDNA haplogroup. Nevertheless, the basic trends of the mtDNA haplogroup distributions are consistently recovered even from the trace amounts of human mtDNA found in our samples.

### Reproducibility

To obtain a general idea about the accuracy and reproducibility of our analysis results, four different samplings of the same city but different wastewater treatment plants (El Paso) were plotted alongside each other in Supplementary Fig. 7., and two different sites (Kitwe and Lusaka) from Zambia in Supplementary Fig. 6. All these samples were treated as non-related during the whole analysis pipeline. Simply comparing the pie charts by visual inspection, the results are strikingly similar for all samples collected at the same site or geographically near to each other. Given that mtDNA haplogroups H and V are remarkably close to each other on the phylogenetic tree of human mitochondrial haplogroups^29^, the pie charts of El Paso become all the more alike.

This suggests that reconstructing the local human mtDNA haplogroup frequencies from sewage samples using the proposed pipeline not only produces results that are in line with previously published data, but that are also highly robust and reproducible.

## Discussion

Many previous studies on human populations have focused on determining the original native populations or ancient evolution and migration, thus, ignoring as much as possible the subpopulations of minorities, temporary foreign workers, immigrants or tourists who may in some places outnumber the residents. Data describing the actual population composition are however, important to study demographic tendencies, health and related socioeconomic trends^45^ and would be a valuable asset for various institutions and organizations, allowing greater efficiency in the provision of services, support and in improving preventive interventions^11^. On the other hand, the introduction of ethnic monitoring is a politically sensitive issue that usually evokes resistance and many feel, that collecting data might itself be discriminatory. Handling such sensitive data by governments and other organizations brings up further questions from fear of racial discrimination of ethnic groups to data security. Collection of detailed information on the genetics of the population may provide additional benefits for public health policy makers but such attempts may face an even stronger resistance.

In this study we provide evidence that by short read sequencing of urban sewage the local composition of human populations in respect to their mtDNA haplogroups in a sewage catchment area can be robustly determined and is in reasonable agreement with the available data. Although the main focus of our analysis was the identification of mtDNA haplogroups, the same method with minor modification in the sampling process might be feasible to recover genotype distributions as well. This presents a great possibility for future studies of ethnic and genetic composition of populations, given that these types of analyses are non-invasive, require no informed consent, do not suffer from the limitations of self-reporting and by their nature, provide a well-mixed sampling of the local population.

Many studies indicate that different mitochondrial DNA haplogroups are variously associated with medical conditions and genetic diseases. These include coronary artery disease, diabetic retinopathy, early-onset Alzheimer’s disease, frontotemporal lobar degeneration, AIDS progression, breast, prostate and renal cancer and many more^46–50^. Many of these established associations are already applied in clinical practice as either biomarkers or aids for patient stratification^51^. These findings suggest that a comprehensive investigation of the mtDNA haplogroup composition of populations at different geographical locations could serve as a helpful guide for disease control by allowing for region-specific prevention strategies and increasing awareness of medical conditions more likely to occur in the local society. Our results demonstrate that the sequencing of urban sewage followed by a subsequent analysis using our proposed pipeline could not only make such a project feasible, but also produce reliable and accurate results.

Population level collection of complete human genome sequences can be even richer source of genetics related health monitoring. As the DNA purification method used for our samples was optimized for the isolation of bacterial DNA^52^, our results suggest that by further optimization to target human DNA, an increased sequencing depth would provide an even more detailed view of population genetics. Many genetic diseases are associated with the presence of specific single nucleotide variations or insertions/deletions that have been shown to occur non-uniformly across different populations^53–55^. Thus, by analysing the frequencies of these mutations from sewage samples, purposeful steps could be taken to ensure locally effective screening and prevention. Mapping out the genetic landscape of different populations with a city-scale, or even larger resolution would also be beneficial for resolving ancestry-related questions and aiding forensics efforts. Similarly, population genetic studies are commonly based on genetic data from a large number of individuals without the explicit need for personal identification, thus retrieving whole genome sequences from urban sewage would be ideal for this purpose.

Emerging technologies like blockchains^56^ promise complete anonymity even for genomic data, but it may take decades to build trust for such systems for the general population. Thus, in contrast to individual level whole genome analysis, sequencing population level DNA mixtures from sewage may provide a viable path. Even though it has been previously shown^14^ that the presence or absence of a single individual can be established even at a trace level from a pooled mixture of various DNAs, the complete genome of the person of interest has to be on hand prior to the analysis. Thus pooled community sequencing does not contribute an additional risk of possible violations of privacy for individuals whose genome is otherwise unknown. The fact that the sequencing of sewage samples requires no active participation from the community makes the technique even more appealing.

Our results also highlight the future possibility of monitoring demographic effects (such as global migration or the segregation of local communities) in the population in-time, as wastewater collection can be accomplished without the need for lengthy preparations and high cost investments and thus can be repeated as required.

It should be emphasized that the data analysed for this study was collected for the purpose of studying the global distribution and abundance of antimicrobial resistance genes and not human genetics. Thus, potential future studies on human populations based on sewage should take into consideration the specific features of the sewage catchment area; among others their exact geographic location in the individual cities and countries, to make sure that a representative sampling of the local population is achieved by wastewater collection. Our results do however, show the potential of analysing urban sewage not only for antimicrobial resistance and infectious disease agents, but also human populations in one and the same analysis.

## Methods

### Sample acquisition

Urban sewage was collected globally from 79 sample locations, covering seven geographical regions from 74 cities in 60 countries^17^. DNA was extracted from the sewage pellets according to an optimized protocol using the QIAamp Fast DNA Stool Mini Kit including twice the input material and initial bead beating^57^. DNA from all samples was mechanically sheared to a targeted fragment size of 300 bp using ultrasonication (Covaris E220evolution). Library preparation was performed with the NEXTflex PCR-free Library Preparation Kit (Bioo Scientific). The Bioo NEXTflex-96 adapter set (Bioo Scientific) was used, and in batches of roughly 60 samples, the libraries were multiplexed and sequenced on the HiSeq. 3000 platform (Illumina), using 2 × 150-bp paired-end sequencing per flow cell with a mean of 120 million reads (range: 8 to 398 million) per sample.

### Identification of human reads

Short reads were aligned to the reference genome with the BWA-MEM^58^ algorithm. The human mitochondrial revised Cambridge Reference Sequence^59^ (NCBI ID: NC_012920.1) was used as a reference genome and the default settings were used to conduct the alignment. To lower the risk of misinterpreting the results, and to verify that the reads mapped to the human mtDNA were indeed derived from humans, we also performed an alignment to several vertebrate species (*Bos taurus*, *Sus scrofa*, *Danio rerio*, *Canis lupus familiaris*, *Gallus gallus*, *Ovis aries;* data not shown) including the Norway rat (*Rattus norvegicus*) mitochondrion sequence (NCBI ID: NC_001665.2) (see Fig. 2b).

Alignment results were validated for the presence of PCR duplicates with the samtools software tool^60^, but none were detected, thus eliminating the need for duplicate removal. Sewage samples collected at different times but originating from the same treatment plant were pooled together during analysis to obtain higher coverage. In cases where samples from the same general geographic location, but from different treatment plants reached the necessary level of coverage, this allowed the comparison of samples collected from the same city (El Paso) or near to each other (Kitwe and Lusaka).

Only 44 samples were considered for further analysis that had an average coverage of at least 10 in the human mitochondrion.

### Principal component analysis

After short read alignment, a principal component analysis (PCA) was carried out based on the most dominant base (supported by the majority of the aligned reads) found in each genomic position of the mtDNA using the samtools mpileup command^60^. The data for the analyzed 44 samples was condensed into a matrix of shape (44, 4·16,569) using one hot encoding. This was achieved by assigning a value of 1 to the most dominant base in each genomic position for each sample, and a value of 0 to all the other bases. PCA was performed using scikit-learn^61^ python package.

### t-distributed stochastic neighbor embedding

Using the above described one hot encoded matrix, a t-distributed Stochastic Neighbor Embedding (t-SNE)^34^ pipeline was also run on the data with the scikit-learn^61^ python module. As suggested by the manual, initial dimension reduction was achieved by selecting the top 50 most dominant PCA components of the originally 4·16,569-dimension space and t-SNE was performed in a subsequent step.

### Phylogenetic tree construction

To obtain a general idea about how the samples might relate to each other, we performed a simple phylogenetical analysis. As a first step, consensus sequences were generated for each sample with the help of bcftools and vcfutils. These are more refined than the above described method of simply choosing the most common base at each genomic position in the sense, that if multiple bases were found at a given position, it is also possible to assign the somewhat ambiguous “pyrimidine” or “purine” values to these sites. These sequences were then multiple aligned with the ClustalW algorithm implemented in Biopython^62^. Phylogenetic trees were constructed with two different methods (neighbor joining^63^ and maximum parsimony^64^) using the Phylo module of Biopython. The robustness of the trees was accessed as the proportion of the 1000 phylogenetic trees created with bootstrapping that agreed with the topology of the original tree for each clade separately.

### Reconstruction of contributions from different mtDNA haplogroups

Given that human mtDNA sequences present in sewage samples are likely to be diverse mixtures of general populations living in a specific area, it can be of great interest to decompose the available reads to differently weighted contributions of various mtDNA haplogroups. To achieve this, we used a computational tool called mixemt^37^. The algorithm uses the database provided by PhyloTree.org (Phylotree Build 17) that describes the defining mutations of over 5000 mtDNA haplotypes. After aligning short reads of the investigated samples to the reference sequence of the human mitochondrion, the pipeline assigns a value to each read and mtDNA haplotype pair that describes how consistent the variants in the given read are with the given mtDNA haplotype, while accounting for sequencing errors. It also sets the initial haplotype proportions in the sample randomly by drawing from a Dirichlet distribution. Then it employs an expectation maximization approach, which first calculates the conditional probabilities of observing the variants in the read in the mtDNA haplotype, given the current mixture proportions. The new mixture composition is determined by finding the values that maximize the conditional probabilities. These steps are iterated until convergence. Once convergence is reached, the mtDNA haplotypes present in the sample are reevaluated by employing additional filtering steps and the iteration is then repeated on the contributing mtDNA haplotypes only, resulting in the final mtDNA haplotype composition of the sample. We used default settings with the -V option, as suggested for low coverage samples. Identified mtDNA haplotypes were grouped into mtDNA haplogroups for easier comparison with available data.

### Comparison with available mtDNA haplogroup composition data

Relevant information about the mtDNA haplogroup distribution of the investigated cities was gathered from literature. We aimed to compare our data with city-, or region-specific results of different studies whenever possible, however, in the absence of such particular information, the country level distributions were used for reference.

Given the lack of specific mtDNA haplogroup distribution data for US cities, we collected census data available about the ethnic composition of the cities and used the results of Just *et al*.^65^ to convert these ratios to a distribution of mtDNA haplogroups belonging to four broad biogeographic ancestry categories. These categories are indicated by different colors of the underscores for each haplogroup on Fig. 3. Since Just *et al*.^65^ contained no specific information about the mtDNA haplogroup composition of the Asian population in the US, the crude assumption was made that individuals with self-reported Asian ethnicity belong strictly to the Asian ancestry group. Even though this obvious simplification might skew the results, given the relatively low ratio of Asian population in US cities, this effect is presumed to be negligible. The mtDNA haplogroup composition obtained from sewage data was also transformed to ratios of the four main ancestry categories for more direct comparison in the case of US cities (Supplementary Fig. 7.).

### Materials & correspondence

Correspondence and material requests should be addressed to István Csabai.

## Supplementary information

Supplementary information

## Data Availability

Sequencing data analysed by this study were collected and prepared by Hendriksen *et al*.^17^ and can be found on the European Nucleotide Archive (ENA; http://www.ebi.ac.uk/ena/) under study accession number ERP109094.
