# Supplementary material for: Worldwide human mitochondrial haplogroup distribution from urban sewage
Source: Sci Rep. 2019 Aug 12;9:11624. doi: 10.1038/s41598-019-48093-5 (PMC6690936; doi:10.1038/s41598-019-48093-5)
Supplement: Supplementary file 1 — Supplementary information [file 41598_2019_48093_MOESM1_ESM.docx]

Supplementary Information for

Worldwide human mitochondrial haplogroup distribution from urban sewage

Orsolya Anna Pipek, Anna Medgyes-Horváth, László Dobos, József Stéger, János Szalai-Gindl, Dávid Visontai, Rolf S. Kaas, Marion Koopmans, Rene S. Hendriksen, Frank M. Aarestrup, István Csabai

Corresponding author: István Csabai

Email: [csabai@complex.elte.hu](mailto:csabai@complex.elte.hu)


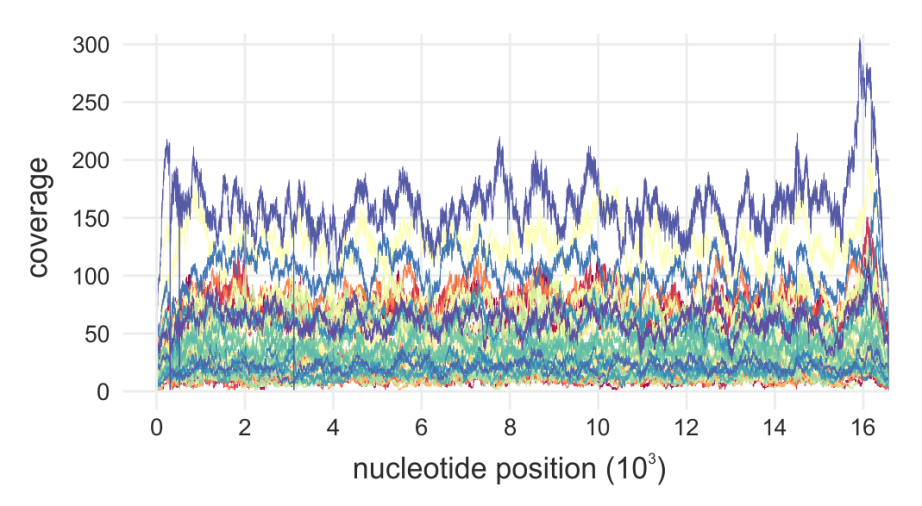


Supplementary Figure 1. Coverage of the human mtDNA in investigated samples. Coverage along the length of the human mtDNA for the 44 samples with an average coverage of 10 or higher. Each colored line represents a single sample. The approximately uniform distributions indicate that the chances of misaligned non-mtDNA reads are minimal.


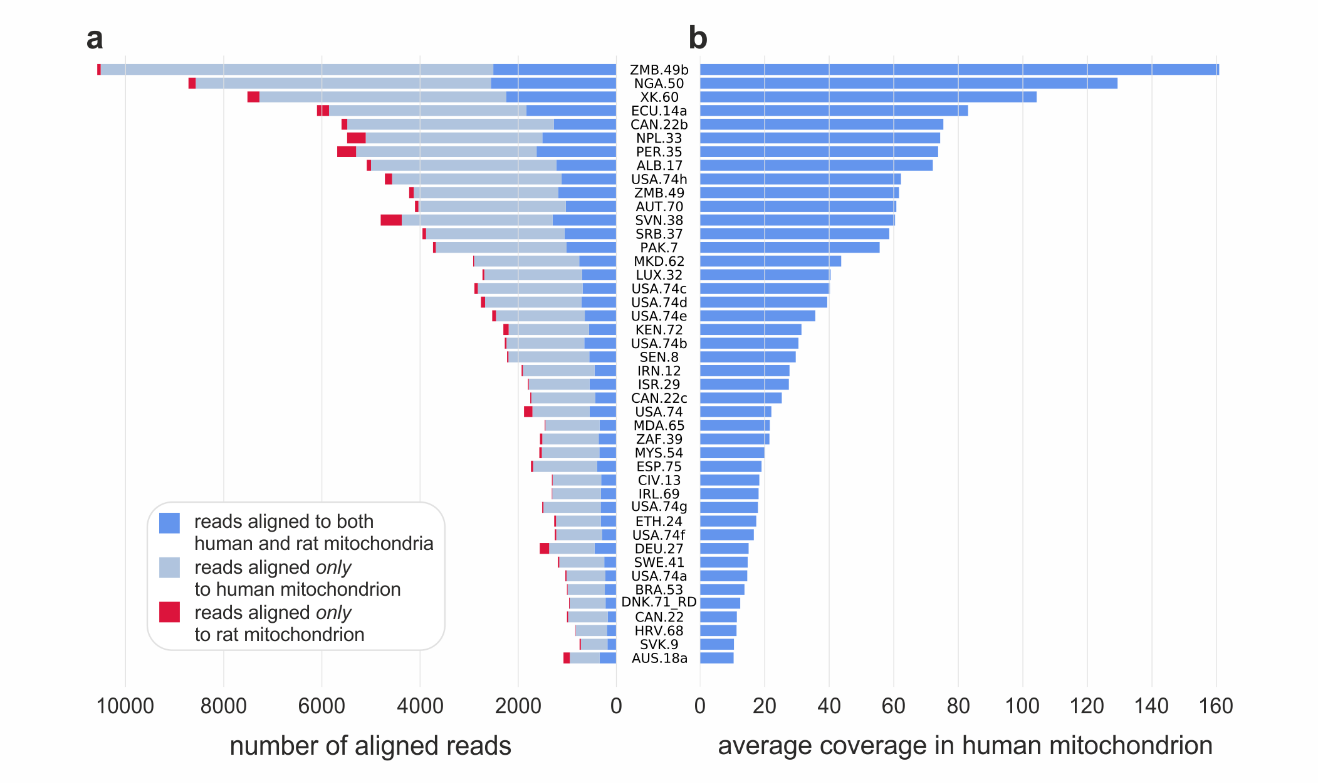


Supplementary Figure 2. Results of short read alignment in sewage samples. a. Number of reads aligned to both and unique to either the human and rat mitochondria in the 44 sewage samples with an average coverage of the human mitochondrion of 10 or higher. b. Average coverage of the human mitochondrion in the 44 samples with an average coverage of 10 or higher. Samples are referenced by their IDs (for more details, see Supplementary Table 1).


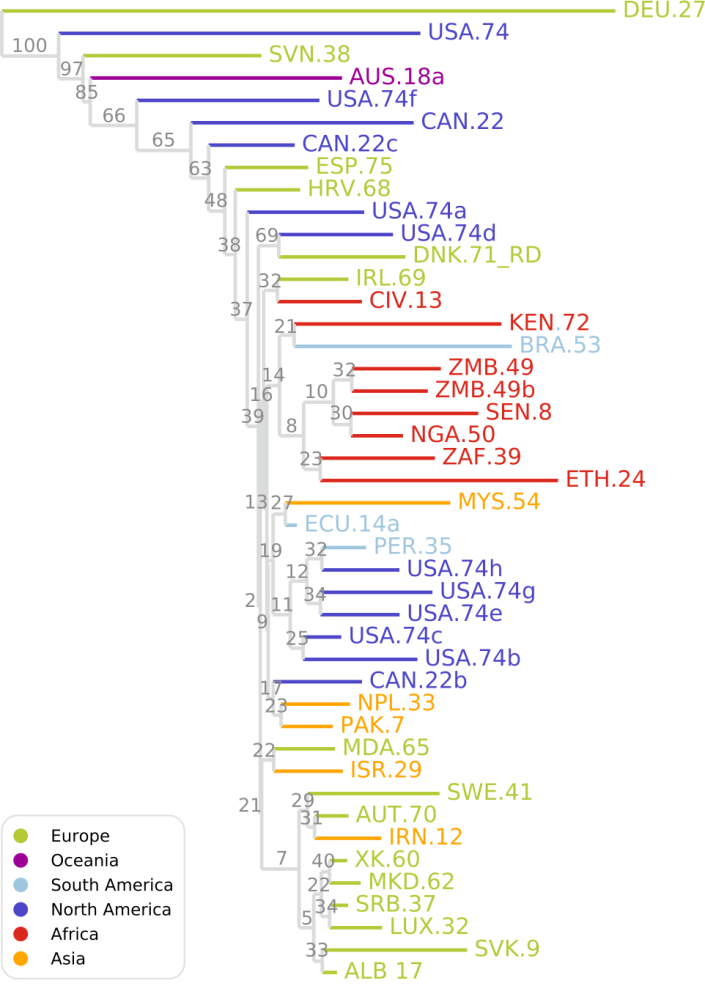


Supplementary Figure 3. Maximum parsimony phylogenetic tree for the samples with an average coverage of 10 or higher with 1000 bootstraps. Grey numbers indicate bootstrap confidence levels (%). Different originating continents are marked with different colors. Samples are referenced by their IDs (for more details, see Supplementary Table 1).


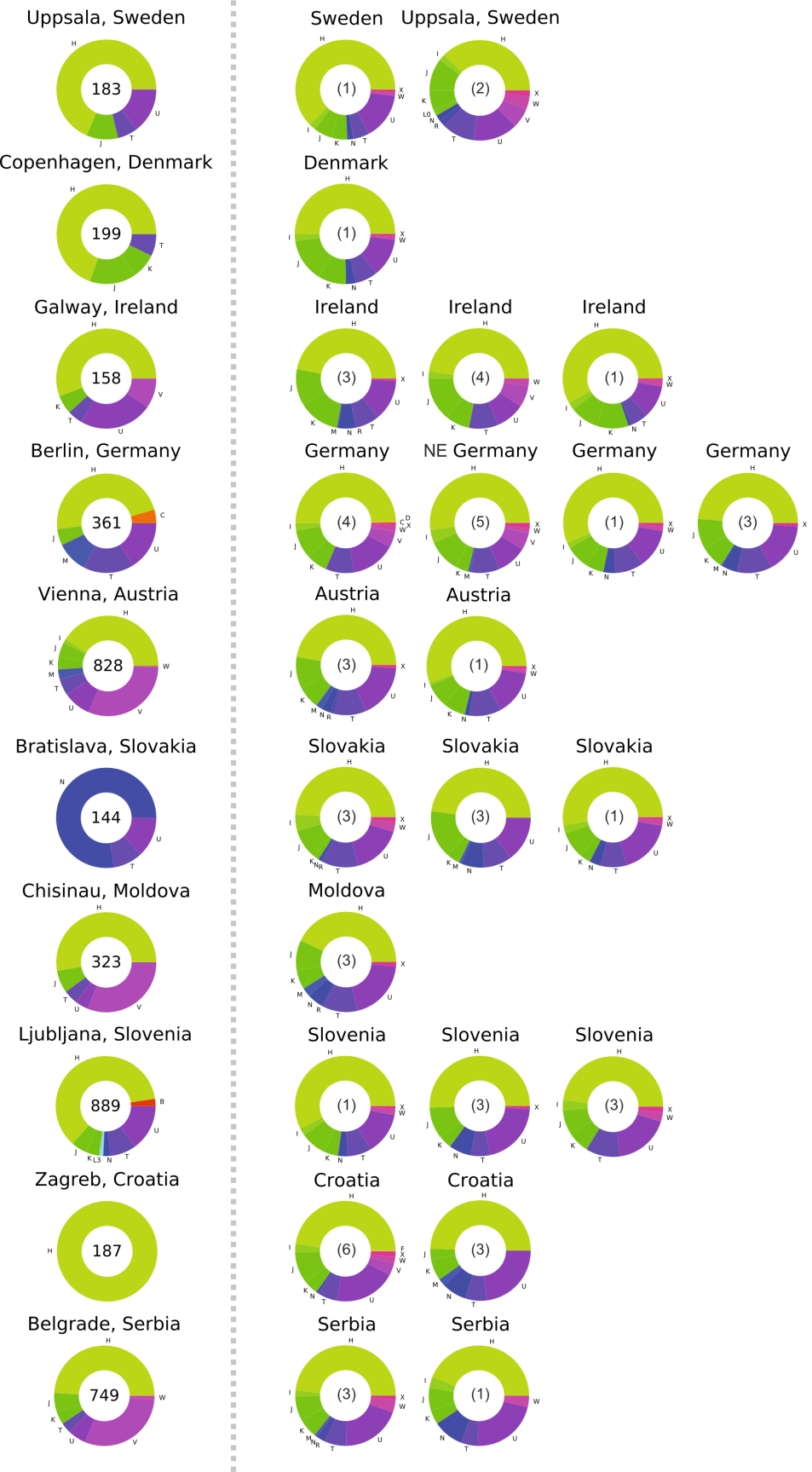


Supplementary Figure 4. mtDNA haplogroup composition of Eurasian cities determined from sewage samples (left of dashed line) compared with data from literature (right of dashed line). Numbers in the middle of pie charts on the left of the dashed line indicate the total number of reads successfully categorized to either mtDNA haplogroup. Numbers in brackets in the middle of the pie charts on the right of the dashed line indicate references to where the data was obtained from. References are listed at the end of the document. (NE: Northeast)^1–6^


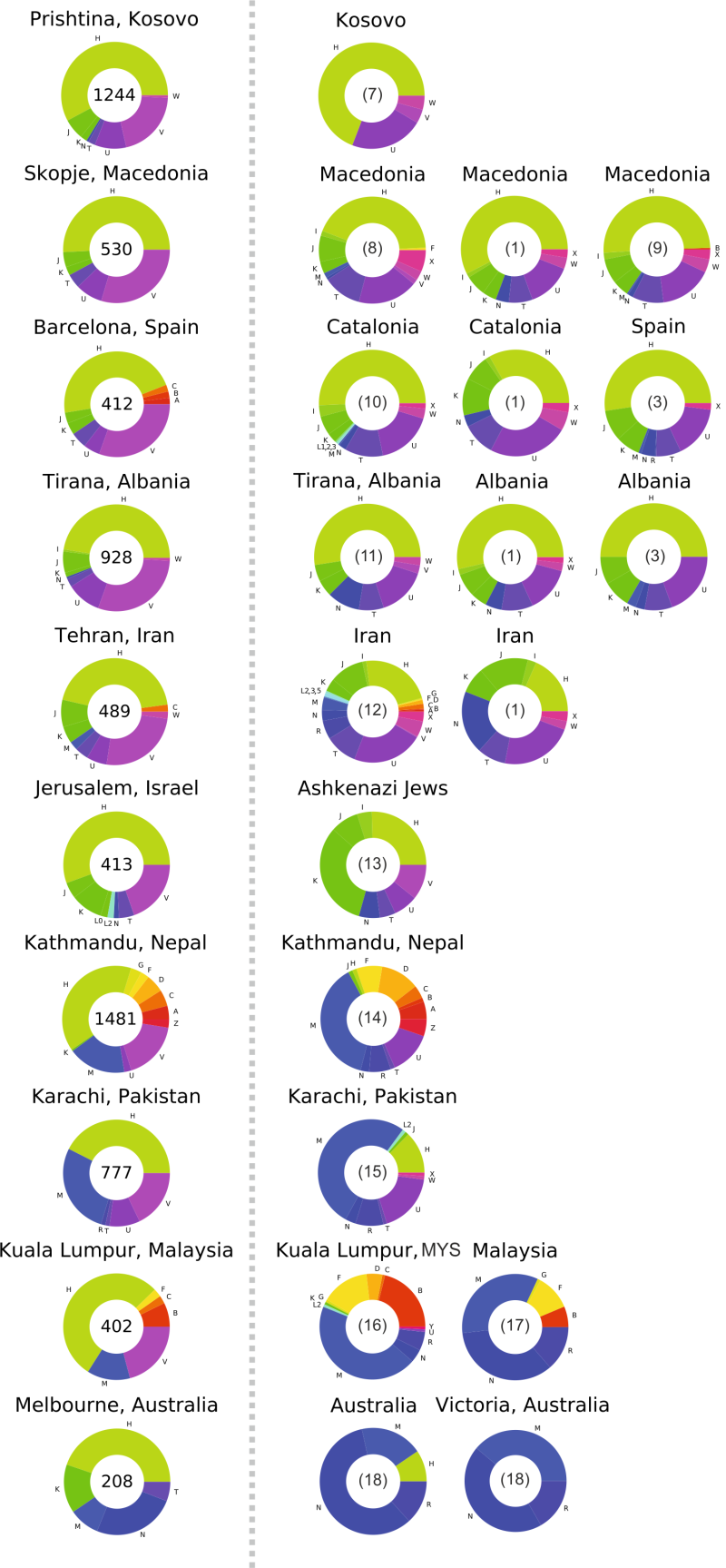


Supplementary Figure 5. mtDNA haplogroup composition of Eurasian cities and Melbourne determined from sewage samples (left of dashed line) compared with data from literature (right of dashed line). Numbers in the middle of pie charts on the left of the dashed line indicate the total number of reads successfully categorized to either mtDNA haplogroup. Numbers in brackets in the middle of the pie charts on the right of the dashed line indicate references to where the data was obtained from. References are listed at the end of the document. (MYS: Malaysia)^1,3,7–18^


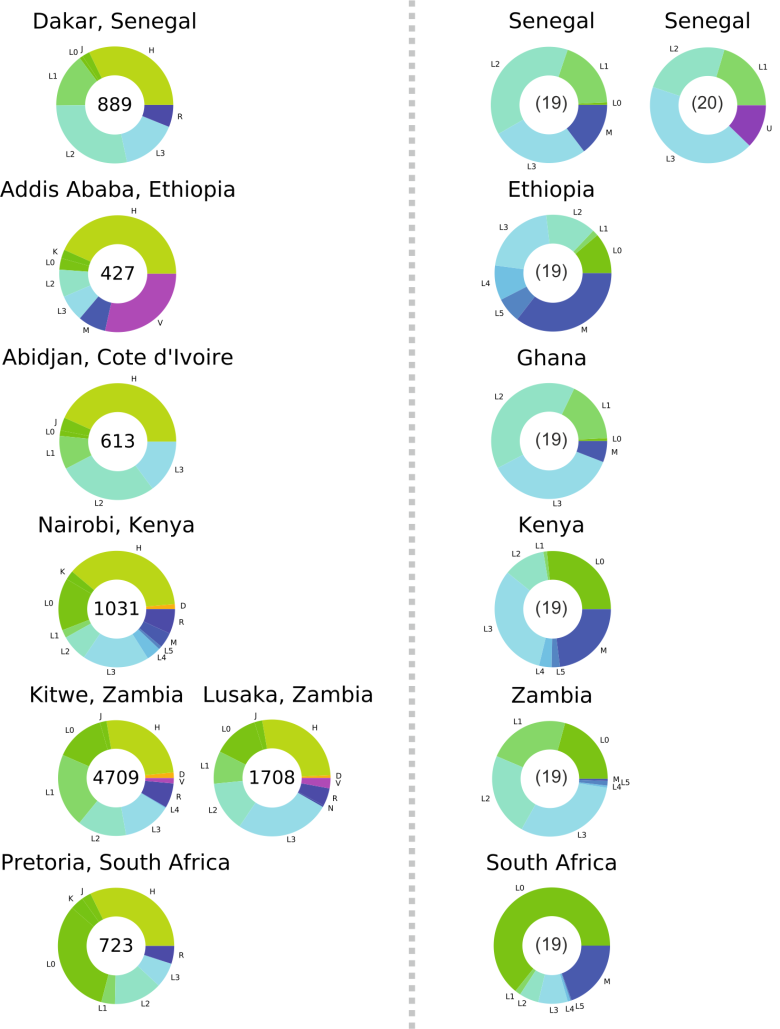


Supplementary Figure 6. mtDNA haplogroup composition of African cities determined from sewage samples (left of dashed line) compared with data from literature (right of dashed line). Numbers in the middle of pie charts on the left of the dashed line indicate the total number of reads successfully categorized to either mtDNA haplogroup. Numbers in brackets in the middle of the pie charts on the right of the dashed line indicate references to where the data was obtained from. References are listed at the end of the document.^19,20^


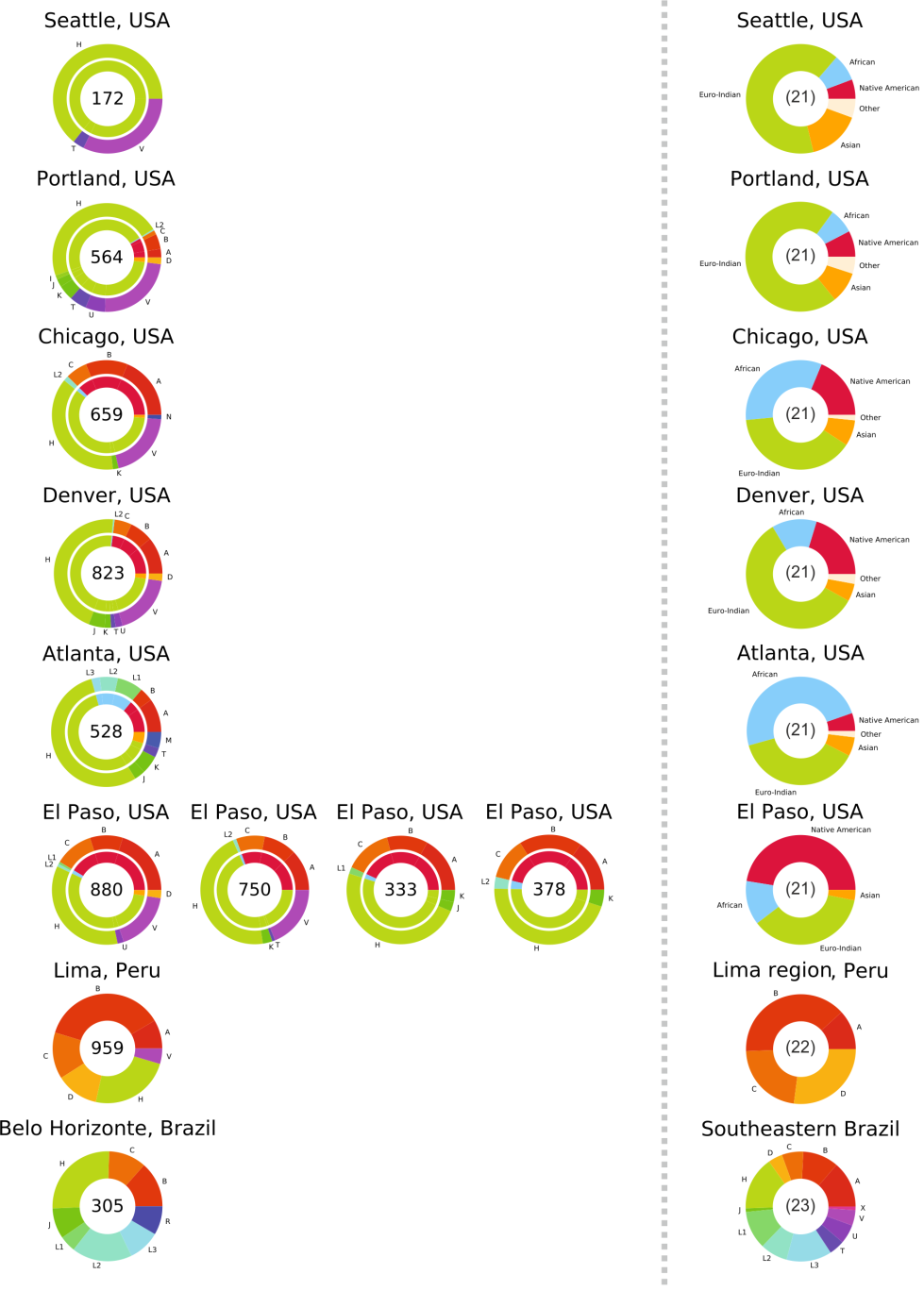


**Supplementary Figure 7.** **mtDNA haplogroup composition of North and South American cities determined from sewage samples (left of dashed line) compared with data from literature (right of dashed line).** Haplogroups for US cities were assigned to one of four broad biogeographic ancestry categories on the inner pie charts for easier comparison with census data (see Materials and methods). Numbers in the middle of pie charts on the left of the dashed line indicate the total number of reads successfully categorized to either mtDNA haplogroup. Numbers in brackets in the middle of the pie charts on the right of the dashed line indicate references to where the data was obtained from. References are listed at the end of the document.^21–23^

Supplementary Table 1. Sample IDs and wastewater collection sites for the 44 samples with average coverage higher than 10 in the human mitochondrion.

| **Sample ID** | **Wastewater collection site (city, country)** |
| --- | --- |
| ALB.17 | Tirana, Albania |
| AUS.18a | Melbourne, Australia |
| AUT.70 | Vienna, Austria |
| BRA.53 | Belo Horizonte, Brazil |
| CAN.22b | Toronto, Canada |
| CAN.22c | Ottawa, Canada |
| CAN.22 | Regina, Canada |
| CIV.13 | Abidjan, Cote d'Ivoire |
| DEU.27 | Berlin, Germany |
| DNK.71_RD | Copenhagen, Denmark |
| ECU.14a | San Cristobal Island, Ecuador |
| ESP.75 | Barcelona, Spain |
| ETH.24 | Addis Ababa, Ethiopia |
| HRV.68 | Zagreb, Croatia |
| IRL.69 | Galway, Ireland |
| IRN.12 | Tehran, Iran |
| ISR.29 | Jerusalem, Israel |
| KEN.72 | Nairobi, Kenya |
| LUX.32 | Luxembourg-city, Luxembourg |
| MDA.65 | Chisinau, Moldova |
| MKD.62 | Skopje, Macedonia |
| MYS.54 | Kuala Lumpur, Malaysia |
| NGA.50 | Lagos, Nigeria |
| NPL.33 | Kathmandu, Nepal |
| PAK.7 | Karachi, Pakistan |
| PER.35 | Lima, Peru |
| SEN.8 | Dakar, Senegal |
| SRB.37 | Belgrade, Serbia |
| SVK.9 | Bratislava, Slovakia |
| SVN.38 | Ljubljana, Slovenia |
| SWE.41 | Uppsala, Sweden |
| USA.74a | Seattle, USA |
| USA.74b | Chicago, USA |
| USA.74c | El Paso, USA |
| USA.74d | Portland, USA |
| USA.74e | El Paso, USA |
| USA.74f | El Paso, USA |
| USA.74g | El Paso, USA |
| USA.74h | Denver, USA |
| USA.74 | Atlanta, USA |
| XK.60 | Pristina, Kosovo |
| ZAF.39 | Pretoria, South Africa |
| ZMB.49b | Kitwe, Zambia |
| ZMB.49 | Lusaka, Zambia |

**References**

1. Maciamo. Eupedia.

2. Lappalainen, T. *et al.* Population structure in contemporary Sweden - A Y-chromosomal and mitochondrial DNA analysis. *Ann. Hum. Genet.* **73,** 61–73 (2009).

3. Cocoş, R. *et al.* Genetic affinities among the historical provinces of Romania and Central Europe as revealed by an mtDNA analysis. doi:10.1186/s12863-017-0487-5

4. Helgason, A. *et al.* mtDNA and the Islands of the North Atlantic: Estimating the Proportions of Norse and Gaelic Ancestry. *Am. J. Hum. Genet* **68,** 723–737 (2001).

5. Poetsch, M., Wittig, H., Krause, D. & Lignitz, E. Mitochondrial diversity of a northeast German population sample. *Forensic Sci. Int.* **137,** 125–132 (2003).

6. Šarac, J. *et al.* Maternal genetic heritage of southeastern europe reveals a new croatian isolate and a novel, local sub-branching in the X2 haplogroup. *Ann. Hum. Genet.* **78,** 178–194 (2014).

7. Bosch, E. *et al.* Paternal and maternal lineages in the Balkans show a homogeneous landscape over linguistic barriers, except for the isolated Aromuns. *Ann. Hum. Genet.* **70,** 459–487 (2006).

8. Derenko, M. *et al.* Complete Mitochondrial DNA Diversity in Iranians. (2013). doi:10.1371/journal.pone.0080673

9. Feder, J. *et al.* Differences in mtDNA haplogroup distribution among 3 Jewish populations alter susceptibility to T2DM complications. *BMC Genomics* **9,** (2008).

10. Gayden, T. Genetic Diversity in the Himalayan Populations of Nepal and Tibet. *FIU Electronic Theses and Dissertations* (Florida International University, 2012). doi:10.25148/etd.FI12042312

11. Quintana-Murci, L. *et al.* Where West Meets East: The Complex mtDNA Landscape of the Southwest and Central Asian Corridor. *Am. J. Hum. Genet* **74,** 827–845 (2004).

12. Jinam, T. A. *et al.* Evolutionary History of Continental Southeast Asians: &quot; Early Train &quot; Hypothesis Based on Genetic Analysis of Mitochondrial and Autosomal DNA Data. doi:10.1093/molbev/mss169

13. Nagle, N. *et al.* Mitochondrial DNA diversity of present-day Aboriginal Australians and implications for human evolution in Oceania. *J. Hum. Genet. Adv. online Publ.* (2016). doi:10.1038/jhg.2016.147

14. Maruyama, S., Nohira-Koike, C., Minaguchi, K. & Nambiar, P. MtDNA control region sequence polymorphisms and phylogenetic analysis of Malay population living in or around Kuala Lumpur in Malaysia.

15. Coklo, M. *et al.* Diversity of Y-chromosomal and mtDNA markers included in Mediscope Chip within two Albanian subpopulations from Croatia and Kosovo: Preliminary data. *Coll. Antropol.* **40,** 195–198 (2016).

16. Zimmermann, B. *et al.* Mitochondrial DNA control region population data from Macedonia. *Forensic Sci. Int. Genet.* **1,** 4–9 (2007).

17. Santos, C. *et al.* Mitochondrial DNA and Y-chromosome structure at the mediterranean and Atlantic façades of the Iberian Peninsula. *Am. J. Hum. Biol.* **26,** 130–141 (2014).

18. Cvjetan, S. *et al.* Frequencies of mtDNA Haplogroups in Southeastern Europe. *Coll. Antropol* **28913,** 193–198 (2004).

19. Silva, M. *et al.* 60,000 years of interactions between Central and Eastern Africa documented by major African mitochondrial haplogroup L2. (2015). doi:10.1038/srep12526

20. Stefflova, K., Dulik, M. C., Pai, A. A., Walker, A. H. & Zeigler-Johnson, C. M. Evaluation of Group Genetic Ancestry of Populations From Philadelphia and Dakar in the Context of Sex-Biased Admixture in the Americas. *Am. PLoS ONE* **4,** 1–10 (2009).

21. U.S. Census Bureau. The Demographic Statistical Atlas of the United States - Statistical Atlas. (2015). Available at: https://statisticalatlas.com/. (Accessed: 9th October 2018)

22. Sandoval, J. R. *et al.* The Genetic History of Peruvian Quechua-Lamistas and Chankas: Uniparental DNA Patterns among Autochthonous Amazonian and Andean Populations. *Ann. Hum. Genet.* **80,** 88–101 (2016).

23. Alves-Silva, J. *et al.* The Ancestry of Brazilian mtDNA Lineages. *Am. J. Hum. Genet* **67,** 444–461 (2000).
